# Strength and Wear Behavior of Three-Dimensional Printed and Prefabricated Denture Teeth: An *In Vitro* Comparative Analysis

**DOI:** 10.1055/s-0042-1759885

**Published:** 2023-01-20

**Authors:** Mohammed M. Gad, Haidar Alalawi, Sultan Akhtar, Raghad Al-Ghamdi, Rahaf Alghamdi, Alaa Al-Jefri, Faisal D. Al-Qarni

**Affiliations:** 1 Department of Substitutive Dental Sciences, College of Dentistry, Imam Abdulrahman Bin Faisal University, Saudi Arabia; 2 Department of Biophysics, Institute for Research and Medical Consultations (IRMC), Imam Abdulrahman Bin Faisal University, Saudi Arabia; 3 College of Dentistry, Imam Abdulrahman Bin Faisal University, Saudi Arabia

**Keywords:** 3D printing, prefabricated teeth, complete denture, mechanical testing

## Abstract

**Objectives** With advanced technology for complete denture fabrication, there is a lack of knowledge on the mechanical behavior of three-dimensional (3D) printed teeth despite the development of complete denture fabrication technologies. This study aimed to compare different types of 3D-printed teeth in terms of wear and fracture resistance in comparison to control prefabricated denture teeth.

**Materials and Methods** One prefabricated tooth was selected and fixed in a resin holder and half of the tooth remained in anatomic form, while the other half was flattened for the wear test. One from each type was scanned and then printed with different resins; Asiga (DentaTOOTH, Asiga, Alexandria 2015,NSW, Australia), FormLabs (Denture Base LP, FormLabs, Berlin, Germany), and NextDent (NextDent C&B MFH, NextDent B.V., Soesterberg, the Netherlands) according to manufacturer recommendations. A total of 60 specimens (20/resin, *n* = 10) were thermo cycled (5,000 cycles) and wear test samples were further subjected to cyclic loading (1,70,000 cycles) in a chewing simulator machine CS-4.2 (SD Mechatronik GmbH, Germany). The fracture strength of anatomic teeth was measured using a universal testing machine (Instron model 5965, Massachusetts, United States), while Geomagic Control X software was used to assess the amount of wear of flattened teeth. Statistical analyses were performed with one-way analysis of variance with Tukey's post hoc test at significance level of α = 0.05.

**Results** NextDent specimens showed the greatest volume loss, whereas FormLabs specimens showed the least volume loss. Comparing NextDent specimens to FromLabs specimens, FromLabs showed statistically significantly less volume loss ( *p* < 0.001). No other group pairs differed significantly from one another in terms of volume loss ( *p* > 0.06).

**Conclusion** 3D-printed denture teeth showed comparable strength and wear resistance with the prefabricated denture teeth and were suitable for long-term clinical usage except for NextDent that significantly showed the lowest fracture resistance.

## Introduction

Digital dentures are fabricated by subtractive methods or additive manufacturing methods. In the additive method, the photo-polymerized fluid resin is used to fabricate removable prostheses fabrication in layering technique. 1 2 The cost of a printer is lower than that of a milling machine, allowing for broad use. When compared with subtractive milling, printing wastes less material. 3 4 Multiple dentures can be made at once using printing. 3 Complex patterns can be produced using printing but milling occasionally has that limitation. 2 In this technology, Three-dimensional (3D)-printed teeth were printed separately and then bonded to the socketed printed pink denture base resin. 2 Denture teeth can be printed using a variety of technologies, although the two most frequently suggested for denture fabrication are stereolithography (SLA) and digital light processing (DLP). The photosensitive resins were polymerized with a laser beam in the SLA system while a projector provides energy in the DLP system. 5

Prefabricated acrylic resin denture teeth have traditionally been used in the fabrication of dentures. The wear resistance of acrylic resin denture teeth has been improved by manufacturers utilizing a variety of monomers, crosslinking agents, organic and inorganic fillers, coupling agent modification, and surface modification methods. 6 7 These trials yielded conflicting results about whether the modifications enhanced the wear resistance of acrylic resin denture teeth. 6 7

For denture longevity, strength, and occlusal wear resistance are crucial factors. 8 The denture teeth fracture is still the main and annoying problem for both patients and prosthodontists. 9 Gad et al investigated the fracture resistance of one type (NextDent) of 3D-printed teeth and found that 3D-printed resin teeth have high fracture resistance than the prefabricated teeth but this strength was decreased after thermal cycling. 10 Furthermore, Chun et al 9 evaluated the fracture resistance of one type (Dentca) of 3D-printed resin teeth and found that stated that 3D-printed resin teeth are comparable to prefabricated denture teeth. Teeth with high wear resistance are required because teeth wear adversely affected occlusal unit stability, function, and esthetics. 11 Recently, Cha et al 12 studied the wear resistance of one type of 3D-printed teeth and found that 3D-printed denture teeth showed comparable results when compared with prefabricated ones in terms of wear resistance.

In the literature, numerous tooth wear evaluation methods have been reported, including direct cusp height measuring, image analysis, scanning electron microscopy (SEM), computer graphics, and profilometry. 13 14 15 However, such methods are difficult to use and require the measurement of dental casts as well as the examiner's subjective assessment of wear. 16 Furthermore, the existence of numerous tooth wear indexes complicated the standardization, quantification, and reliability of tooth wear quantification, making it impossible to compare the results of different investigations. 17 With the advancement of digital dentistry, 3D scanning has become the preferred method for determining wear. This method is precise and quantitative, and it can be used in both clinical and laboratory studies. It also allows for the storage of 3D databases that can be compared with other 3D databases. 18

With advanced digital technology for denture base fabrication, few studies evaluated the strength of 3D-printed denture teeth. Therefore, further investigations are required to prove their clinical applicability. This study was done to evaluate and compare the fracture resistance and wear resistance of different brands of 3D-printed denture teeth in comparison to the prefabricated denture teeth. The null hypothesis was that no significance in wear and fracture resistance between 3D-printed resins and prefabricated ones.

## Materials and Methods

Three types of photopolymerized fluid resins for denture teeth (FormLabs, Asiga, NextDent) were selected to fabricate a total of 60 specimens (20/resin; 10 anatomic/fracture resistance, and 10 flat for wear resistance). While 20 prefabricated teeth were prepared as control (10 anatomic and 10 flattened for wear resistance. The number of samples per group was determined based on a previous study. 10

A prefabricated mandibular molar tooth (Major Dent-V, MAJOR Prodotti Dentari S.P.A., Moncalieri (TO), Italy) was selected and then fixed in an autopolymerized resin cubic shape base. The teeth cubic base had four angles used to insure perfect alignment of both scans. Ten samples were remaining anatomic, while the others 10 samples were flattened using an abrasive disc mounted on an automated polishing machine (Metaserv 250 grinder-polisher; Buehler GmbH). Flatting of the samples was standardized using a reduction jig (PATTERN RESIN LS, GC America Inc., Illinois, United States). One anatomic tooth and one flat tooth were scanned using a desktop laser scanner (E3; 3Shape A/S, Copenhagen, Denmark) to form an STL file. The STL files were exported to each printer for denture teeth printing. Printed resins, printers, and printing parameters were summarized in Table 1 .

**Table 1:** Printed resins, printers, and printing parameters

3D-printed resin	Manufacturer	Composition	Printer	Printing technology	Printing parameter
NextDent C&B MFH (Micro Filled Hybrid)	NextDent B.V. Centurionbaan 190 3769 AV Soesterberg, the Netherlands	7,7,9(or 7,9,9)-trimethyl-4,13-dioxo-3,14-dioxa-5,12diazahexadecane-1,16-diyl bismethacrylate, 2-hydroxyethyl methacrylate, Silicon dioxide, diphenyl (2,4,6-trimethyl benzoyl) phosphine oxide, ethoxylated bisphenol A dimethacrylate, ethylene dimethacrylate, titanium dioxide, and mequinol; 4-methoxyphenol; hydroquinone monomethyl ether	NextDent 5100	DLP	• Printing angle: 0-degree • Layer thickness: 50µm • Post-curing oven: LC-3DPrint Box • Post-curing time/Temp: 30 minutes/60°C
Asiga DentaTOOTH	(DentaTOOTH, Asiga, 2 / 19-21 Bourke Road, Alexandria 2015, NSW, Australia)	7,7,9(or 7,9,9) trimethyl-4,13-dioxo3,14-dioxa-5,12diazahexadecane-1,16diyl bismethacrylate, tetrahydrofurfuryl methacrylate, and diphenyl(2,4,6trimethylbenzoyl) phosphine oxide	Asiga MAX UV	DLP	• Printing angle: 0-degree • Layer thickness: 50µm • Post-curing oven: Asiga Flash • Post-curing time/Temp: 20 minutes/60°C
Denture teeth resin	FormLabs GmbH Funkhaus Berlin Nalepastr. 18 12459 Berlin, Germany	Bisphenol A dimethacrylate, urethane dimethacrylate, methacrylate monomer, and photoinitiator	FormLabs Form 2	SLA	• Printing angle: 0-degree • Layer thickness: 50µm • Post-curing unit: Form Cure • Post-curing time/Temp: 30 minutes/80°C

Abbreviations: DLP, digital light processing; SLA, stereolithography.

The fluid resins were shaken according to manufacture recommendations and then poured in resin trays for NextDent and Asiga, while FormLabs tank was installed directly to the FormLabs printer. After printing, specimens were cleaned using 99% Isopropyl alcohol to clean unpolymerized resin, and then an additional curing cycle was done to complete the specimens' polymerization. According to manufacturer recommendations, a post-curing oven per resin ( Table 1 ) was used where the specimens per group were immersed in a glycerin bath within the curing unit. 12 All prepared specimens were stored in distilled water for 48 hours at 37°C and then subjected to thermal cycling (5,000 cycles) using a thermocycling machine (SD Mechatronik Thermocycler, Germany) at 5 and 55°C with a dwell time of 30 seconds.

### Wear Resistance Test

All teeth were marked and scanned “Reference Scan” using 3Shape TRIOS 3 scanner (3Shape A/S, Copenhagen, Denmark) and tabulated as baseline readings. For the wear resistance test, a chewing simulator CS-4.2 (SD Mechatronik GmbH, Germany) was used as the method described in a previous study. 10 The previous study employed 60,000 cycles for cyclic loading but suggested increasing it. A greater cycling loading of 1,70,000 cycles was performed to replicate a year of clinical use (∼167,000 cycles). After the wear test, all samples were scanned again “Test Scan” using the same scanner by the same investigator.

**Fig. 1:**
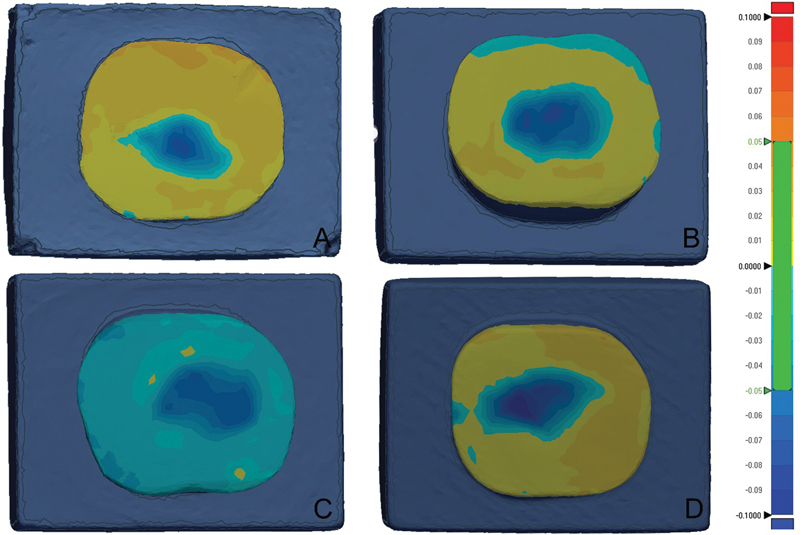
Color mapping showing the different amount of wear in the tested groups—( **A** ) Control, ( **B** ) Asiga, ( **C** ) FormLabs, and ( **D** ) NextDent

Geomagic Control X software (3D Systems, Inc.) was used by one operator to assess the amount of wear. The STL file of the “Reference Scan” and “Test Scan” were imported into the software as “reference data” and “measured data,” respectively. The flat surface on the “reference data” was segmented and selected as the area of comparison. The processes of “Initial alignment” and “Best Fit Alignment” for the “reference data” and “measured date” were completed. Several cross-sectional views were generated to ensure the correct alignment. Then, the process “3D Compare” was operated with a color bar range of ± 0.1mm and tolerance of 0.05 mm ( Fig. 1 ).

The color map displays a spectrum of blue color where wear has occurred, which is indicated by a value on the color bar range. The darker blue indicated a greater volume loss than the lighter blue color. The results report was generated and the “-ve average” value was considered as the volume loss after the wear test.

### Fracture Resistance Test

Specimens were loaded using a universal testing device (Instron model 5965, Massachusetts, United States) at the occlusal surfaces with a stainless-steel ball indenter (7 mm radius) at a loading rate of 1 mm/min to contact the four cusps until failure. 19 To prevent contact damage and contribute to load distribution, a rubber sheet with a thickness of 1.5 mm was placed between the crown and the indenter.

**Fig. 2:**
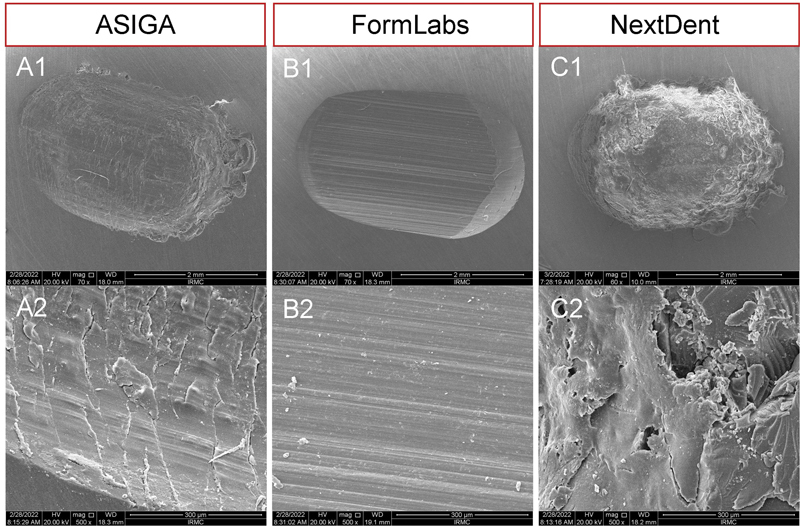
Scanning electron microscopy micrographs (low and high magnifications) of wear patterns of (A1, A2) Asiga, (B1, B2) FormLabs, and (C1, C2) NextDent.

**Fig. 3:**
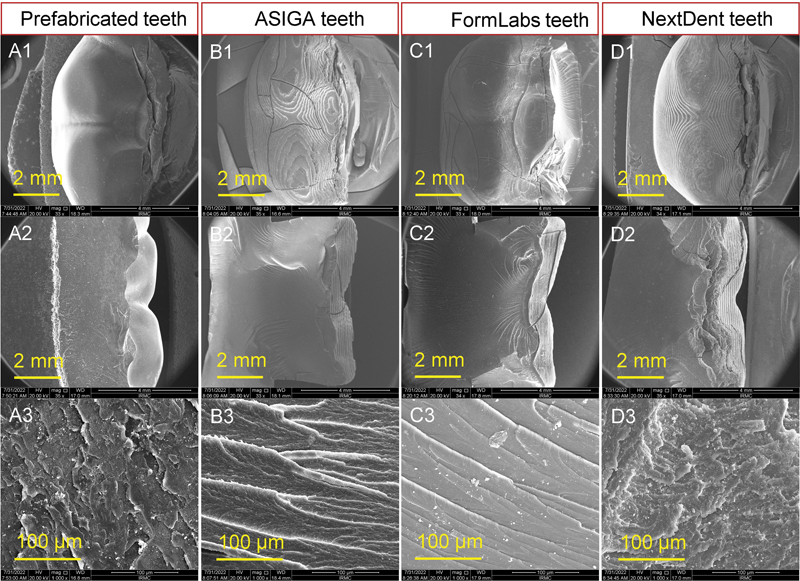
Scanning electron microscopy micrographs of the fractured specimens (prefabricated, Asiga, FormLabs and NextDent teeth)—(A1–D1) Occlusal view, (A2–D2) fracture side full views, and (A3–D3) fracture side enlarged views.

After wear and fracture tests, representative specimen per group was selected for analysis of surface in term of wear patterns and features as well as the mode of teeth fracture. The specimens were gold coated using a sputter coating machine (Quorum, Q150R ES, United Kingdom) and then scanned under SEM (FEI, Inspect S50, Czech Republic, operated at 20 kV). Electronic images were recorded under different magnifications (low and high) to evaluate the required features of the specimens. Fig. 2 showed SEM magnification of x70 (A1 & B1), x60 (C1), and x500 (A2-C2), while Fig. 3 showed SEM magnification of approximately x35 (A1-D1 & A2-D2) and x1000 (A3-D3).

### Statistical Analysis

One-way analysis of variance was used to determine the statistical difference among groups for both wear and fracture resistance test, after normal distribution was confirmed with Shapiro–Wilk test. Pairwise all statistical analyses were performed with Tukey's post hoc test. SPSS (IBM Corp., New York, New York, United States) was used for all statistical analyses with the level of significance set at 0.05.

## Results

The amount of wear was measured as volume loss, which showed a significant difference among groups ( *p* = 0.001). The highest volume loss was observed in NextDent specimens, while FormLabs specimens had the lowest value. Pairwise comparisons showed a significant difference between FromLabs and NextDent specimens ( *p* < 0.001). All other group pairs had similar volume loss, with no significant difference between them ( *p* > 0.06). The mean volume loss for all groups is listed in Table 1 .

Wear behavior of specimens (Asiga, FormLabs and NextDent group) were evaluated by SEM. The wear pattern is illustrated in Fig. 2 . In Fig. 2 , (A–C1), SEM micrographs (low magnifications) showed demarcated outlined depressed area with deep and width, while parts A2-C2 represented the wear surfaces at high magnifications of all groups and displayed compressed and crushed features with some crack lines and grooves except FormLabs group ( Fig. 2B1, B2 ) which displayed fine serrated line with smooth background at the bottom and sides of abraded cavity. While more cracks, microbubbles ( Fig. 2A2 , C2 ), and some voids ( Fig. 2C2 ) were clearly observed in NextDent group.

The results of the fracture resistance test are summarized in Table 2 . Asiga and FormLabs specimens had statistically similar fracture resistance, which was comparable to conventional prefabricated teeth. NextDent specimen, however, had significantly lower fracture resistance than all groups ( *p* <0.001).

**Table 2:** Volume loss (mean and SD [µm]) and fracture load (mean and SD; *n* ) for tested 3D printed resins

Properties	Control	Asiga	FormLabs	NextDent	*p* -Value
Volume loss	31.79 (10.56) ^a,b^	32.30 (6.41) ^a,b^	22.54 (5.9) ^a^	37.55 (5.51) ^b^	0.001*
Fracture load	1421.7 (172.9) ^a^	1305.7 (197.4) ^a^	1285.5 (158.4) ^a^	867.8 (108.4) ^b^	<0.001

Abbreviation: SD, standard deviation.

^a,b^ : Same small letter in each raw indicating statistically insignificant difference between groups. ^*^ *p* -Value less than 0.05 indicate statistically significant difference

**Fig. 4:**
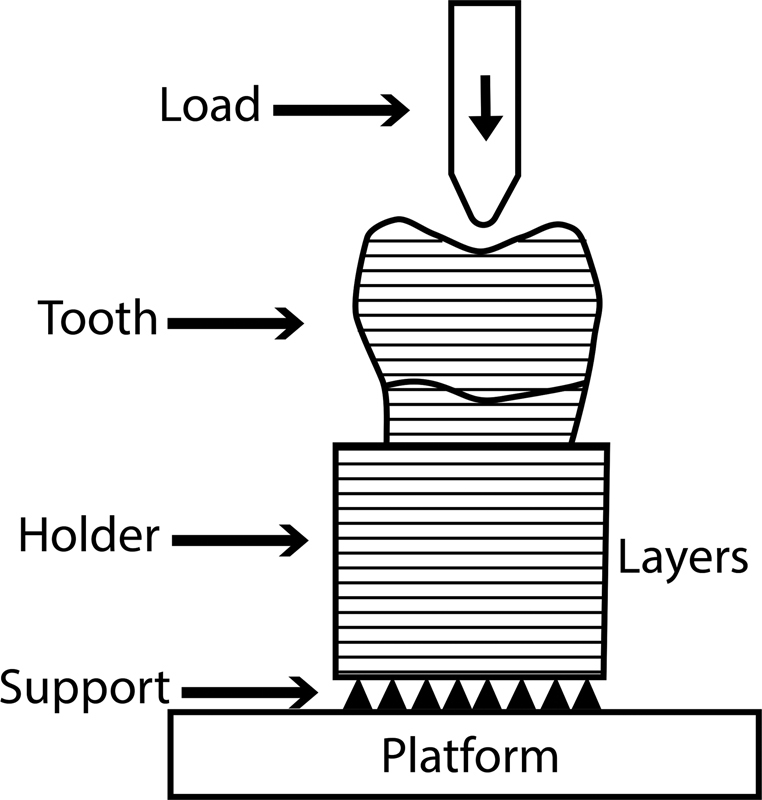
Load direction in relation to printing layer directions.

Regarding SEM findings ( Fig. 3 ) displayed representative SEM images of the fractured specimens, where occlusal view (A1–D1), fracture side view low magnifications (A2–D2), and fracture side view high magnifications (A3-D3) were shown. Generally, the printing layers features (shadow) appear in 3D-printed representative SEM image as the specimens were printed with 0-degree orientation ( Fig. 3B1–D1 ). The prefabricated teeth showed only small cracks at the site of fracture ( Fig. 3A1 ) with smooth fracture side with faint lamellae feature ( Fig. 3A2 ). The high magnification image of the fracture showed small lamella represent a ductile mode of fracture ( Fig. 3A3 ). Some features were presented with Asiga ( Fig. 3B1 –B3) and FormLabs ( Fig. 3C1 –C3) such as crack propagations in different directions when viewed occlusally and addition to the uniform distributed lamellae when viewed from the fracture side ( Fig. 3B2, B3 and Fig. 3C2, C3 ). These features were not obvious with NextDent where the scattered cracks on the occlusal surface were absent ( Fig. 4D1 ) in addition to the smooth side fracture with irregular faint lamellae representing intermediate to brittle fracture ( Fig. 3D2, D3 ).

## Discussion

Although the importance of wear resistance test as indicator for denture teeth longevity, few studies 10 , 12 examined the wear resistance of 3D-printed teeth despite the significance of wear resistance testing as an indicator of the longevity of denture teeth.. Recent review was conducted including these studies and concluded that 3D-printed materials showed promising results compared with prefabricated teeth and showed same behavior before and after chewing simulator effect. 20 However, some resins were equal or close (higher or less) compared with the conventional materials. These variations could be attributed to different brands and different fabrication, opposing type, aging cycles, thermal cycling, and evaluation methods in addition to the different brands of prefabricated teeth. Therefore, comparison of different 3D-printed resin brands under same conditions was hypothesized.

Teeth surface wear was tested by either two-body or three-body wear method. Previous studies used two-body wear type where it displays the effect of direct contact between tested teeth surface and antagonist. 21 In bilateral balanced complete denture occlusion, two-body contact occurs with parafunctional habits and swallowing resulted in teeth wear 10 22 therefore, the two-body wear type was selected for this study. In addition to wear test method, the antagonist materials have an impact on the wear rate of tested resins. 6 In the oral cavity, the removable prostheses are opposed by different materials, denture teeth in complete denture cases, natural teeth, and different restorative materials like zirconia in single denture cases. So, different antagonists (artificial and natural teeth, metal, steel, steatite, ceramics) were suggested in previous studies. 6 10 21 23 24 However, metal was recommended to standardize the wear behavior. 8 10

The load applied to the teeth before testing is an aging method mimicking the oral conditions and it was found that when specimens were loaded for 250,000 cycles, this approximately equals 1 year with natural dentition in the oral cavity. 25 However, complete denture patients are recommended to remove the denture during sleep (∼8 hours per day). When applying this for complete denture occlusion, approximately 167,000 cycles simulate 1 year of clinical use. 7 25 Based on this and the recommendation of the previous study to increase the cycles to more than 40000 cycles, 10 170,000 cyclic loading was applied during the wear resistance test. For the wear test, the estimated force and sliding movement in the chewing simulator were standardized to get reliable *in vitro* results almost comparable to clinical wear. 7 Also, the temperature change in the oral cavity influenced the properties of denture materials, so all specimens were thermally stressed (5,000 cycles). 10 26 While specimens were thermocycled, water uptake occurred and water uptake increased with water temperature. The absorbed water acted as a plasticizer weakening the resins materials. 27 28

There are different methods of digital wear measurements. However, profilometry has been established to be a highly accurate method and might be recognized as the gold standard method to map the surface topography of a particular object. 29 A potential drawback of this research is that an optical scanner was used in place of the profilometry method for this research. White light profilometry and intraoral scanning were compared in a recent study, the results showed that the differences were within the range of predicted measurement error. 30

The null hypothesis stated that no significance in wear and fracture resistance between 3D-printed resin teeth and prefabricated one was partially rejected where Asiga and FormLabs were comparable to prefabricated one, while NextDent was significantly showed lower mechanical performance in comparison to prefabricated teeth.

3D-printed resins exhibited low mechanical performances due to printing nature (additive layering technique) and polymerization methods that were characterized by low degree of conversion and weak bond. 27 31 Additionally, the printing nature; layer-by-layer and interlayers weak bonding contributed to the low mechanical behavior of 3D printed resins. 27 27 It was reported that prefabricated teeth exhibited high wear resistance in comparison 3D-printed resin. 10 The prefabricated teeth were processed under high pressure with different polymerization method and consist of multiple layers with different chemical and physical properties, 23 in addition to the glossy enamel mimic coating layer increasing the wear resistance of prefabricated one as this layer was missing in 3D-printed resins. 10 In this study, 3D-printed resins were comparable to prefabricated teeth; however, the FormLab showed the highest wear resistance (lowest volume loss) value when compared with other groups. This may be due to the printing technology (DLP). The most popular 3D printing processes for creating dental restorations are SLA and DLP, which offer the advantages of excellent precision and quick processing. 24 32 It was reported that printed object with SLA technology showed advantages, good mechanical resistance compared with DLP printed object. 33 34 In a previous study done by Pham et al 23 have investigated the abrasion resistance of 3D-printed denture teeth (FormLabs Resin) in comparison with conventional prefabricated denture teeth and stated that 3D-printed resin exhibited superior abrasion resistance in similarity with findings of the current study. SEM findings proved strength of FormLabs resin; based on SEM finding, FormLabs resin showed less distractive characteristics (crushed, cracks, voids, and microbubbles).

On the other hand, both Asiga and NextDent showed an insignificant decrease in wear resistance in agreement with previous study 10 in compared with conventional prefabricated teeth. Also, another study by Cha et al 12 compared DENTCA 3D-printed resin with different opposing abraders agree with our findings. In similar to Park et al, 24 comparing wear resistance of 3D-printed provisional resin (NextDent C&B) with convectional polymethylmethacrylate (PMMA) conformed no significant differences. In disagreement, Myagmar et al 32 tested the wear resistance of 3D-printed provisional resin (NextDent C&B) and reported that NextDent exhibited lower wear volume loss than conventional provisional resin. This conflict in results may be attributed to the resin type used as a control (provisional resin), while in our study the control was denture teeth (prefabricated one).

Shipping or complete fracture of denture teeth is a common problem occurred with removable prostheses while in clinical use or when subjected to sudden impact force. 9 Hence, the fracture affects the denture longevity, and selection of teeth with high resistance to fracture is recommended. Few studies on 3D-printed teeth have been conducted since digital technology (3D printing) was introduced for denture base fabrication, making it challenging to compare study results. Based on the finding of this study, Asiga and FormLabs showed comparable fracture resistance with conventional prefabricated teeth and showed close mean values to the prefabricated teeth.

A previous study Chung et al 9 investigated different 3D-printed resin (Dentca) and prefabricated teeth and reported same finding of our study. On the other side, NextDent resin showed the lowest fracture resistance when compared with prefabricated one and 3D-printed resins Asiga and FormLabs. NextDent resin teeth showed the lowest fracture resistance with control and other 3D-printed resins. This decrease may be attributed to resins compositions as detailed with wear effects; hence, prefabricated teeth are made of conventional PMMA-based resin; in contrast, 3D-printed NextDent teeth are ester-based resin. 10 In addition to the printing technology, this could be another explanation for decreased strength (SLA printed FormLabs, while Asiga and NextDent are DLP printed technology). This might be because SLA 3D printing is more precise than DLP 3D printing since the resin is cured (hardened) point by point in the SLA printer. Additionally, DLP 3D printers require less time to print than SLA printers, which may reduce the amount of time spent on curing.

In this study, the printing parameters were standardized for all printed resins like 50µm layer thickness and 0-degree printing orientations. 35 In previous studies, 35 36 vertical or horizontal printing orientation were suggested. However, 0 degree was recommended by the manufacturer to make the load applied perpendicular to printing layer orientations as the specimens (full printed teeth no plate or disc specimens) placed on the testing machine with the same printing direction ( Fig. 4 ).

Previous study 10 compared fracture resistance of 3D-printed teeth (NextDent) before and after thermal cycling and found that after thermal cycling the NextDent was comparable to the prefabricated one in disagreement with our finding. The difference may be due to the cyclic loading that increased to 1,70,000 instead of 40,000 cycles and addition of printing orientation 0-degree instead of 90-degree. In this study, the printing orientation was standardized (0-degree). This orientation made the load perpendicular to the printing layer and the vertical force directed to the occlusal surface ( Fig. 4 ) mimicking the clinical conditions. While other orientations (45 and 90 degrees) made the load parallel to the printing layer directions which maybe results in layer separations that affect teeth properties. 36 However, this explanation could be considered with cautions as there are no studies that investigated the effect of printing orientations on denture teeth properties.

The fracture mode is a guide for material strength and two fracture modes were identified: fracture without deformation (cracks-dominant, brittle type) and fracture with deformation (deformation-dominant, quasi-plastic mode). 9 10 37 Regarding the surface characteristics as displayed from SEM analysis of fractured surfaces, prefabricated, FormLabs, and Asiga resin teeth displayed same features (ductile fracture mode). Additionally, the scattered cracks in FormLabs and Asiga suggested that these materials have stronger fracture resistance, also revealed by their higher strength. 38 39 However, NextDent showed different fracture mode that is considered as a sign of low strength in addition to absence of scattered cracks that refer to early material failure with brittle fracture mode.

Clinically, FormLabs and Asiga resins for denture teeth are suitable for clinical as their strength and wear behavior are comparable to the prefabricated teeth. In case of NextDent, further investigations are recommended. Additionally, resin teeth reinforcement with nanoparticles may result in teeth with high strength and more wear resistance like 3D-printed denture base resins reinforced with SiO _2_ and ZrO _2_ nanoparticles. 10 40 By this way, 3D-printed teeth with high strength will be suitable for denture longevity.

Using different brands of 3D-printed resins after aging with more cyclic loading is considered a strength point of this study. However, limitations in this study were having only one antagonist material tested and lack of oral conditions; saliva and its constituents, and the absence of chewing force with different magnitudes and directions. Therefore, *in vivo* testing of the strength and wear behavior of different brands of 3D-printed denture teeth bonded to denture base resins is recommended.

## Conclusion

Although FormLabs resin exhibited less volume loss, all 3D-printed denture teeth showed comparable wear resistance with the prefabricated denture teeth. In terms of fracture resistance, Asiga and FormLabs 3D-printed resin teeth are comparable to the prefabricated teeth and suitable for long-term clinical usage. NextDent significantly showed the lowest fracture resistance.
